# A retrospective study on incidence, diagnosis, and clinical outcome of gastric-type endocervical adenocarcinoma in a single institution

**DOI:** 10.1186/s13000-021-01129-9

**Published:** 2021-07-31

**Authors:** Anna Radomska, Daniel Lee, Heather Neufeld, Nancy Korte, Emina Torlakovic, Anita Agrawal, Rajni Chibbar

**Affiliations:** 1 Department of Pathology and Laboratory Medicine, College of Medicine, University of Saskatchewan, Saskatoon, Saskatchewan Canada; 2 Department of Obstetrics and Gynecology, Queen’s University, Kingston, Ontario Canada

## Abstract

**Background:**

Gastric-type endocervical adenocarcinoma is rare but the most common subtype of cervical adenocarcinoma not associated with human papillomavirus. It is more aggressive with a shorter five-year survival rate compared to human papillomavirus-associated usual type endocervical adenocarcinoma. The objectives of our study were to determine the incidence and clinical-pathological characteristics of Gastric-type endocervical adenocarcinoma in a single institution.

**Methods:**

Twenty four cases of invasive cervical adenocarcinoma were identified between January 2000 and December 2015, from the Saskatoon Health Region pathology database using International Endocervical Adenocarcinoma Criteria and Classification to retrospectively classify endocervical adenocarcinoma. Immunohistochemistry was performed with antibodies for Gastric mucin-6 (MUC-6), p16^INK4a^, cyclin-dependent kinase inhibitor 2A (p16), p53 protein (p53), estrogen and progesterone receptors. Clinical and pathological data was retrieved from pathology reports and charts. Statistical analysis was performed using Mann-Whitney U test and Chi-Square test.

**Results:**

Using the International Endocervical Adenocarcinoma Criteria and Classification criteria, 19 cases (79.2%) were classified as human papillomavirus-associated usual type endocervical adenocarcinoma, and five cases (20.8%) as Gastric-type endocervical adenocarcinoma. In our study 40% of Gastric-type endocervical adenocarcinoma cases presented at stage III compared to none of the usual type endocervical carcinoma cases. All the Gastric-type endocervical adenocarcinoma cases were positive for MUC-6, and negative for p16. 60% Gastric-type endocervical adenocarcinoma cases demonstrated mutant type p53 staining. In contrast, 84.2% of human papillomavirus-associated usual type endocervical adenocarcinoma cases showed block like nuclear and cytoplasmic positivity with p16 antibodies. The Gastric-type endocervical adenocarcinoma group had significantly shorter median survival time than human papillomavirus-associated usual type endocervical adenocarcinoma group, Gastric-type endocervical adenocarcinoma is 22 months compared to human papillomavirus-associated usual type endocervical adenocarcinoma at 118 months (*p* = 0.043).

**Conclusions:**

In this study, Gastric-type endocervical adenocarcinoma accounted for 20.8% of all cervical adenocarcinoma with higher stage at presentation and shorter overall survival. Criteria proposed by International Endocervical Adenocarcinoma Criteria and Classification (IECC) are simple and reproducible in differentiating between, HPV- associated (HPVA) and non HPV associated (NHPVA) endocervical adenocarcinoma. Although none of the IHC assays is specific for GAS, but p16, MUC-6, ER, PR and p53 may further aid in confirming GAS and to differentiate it from benign and malignant mimics.

## Introduction

**Fig. 1:**
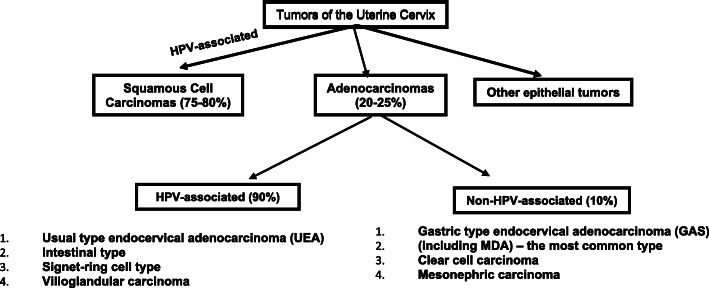
Classification and association of tumors of the uterine cervix

Uterine cervical carcinoma is the fourth most common malignancy in women worldwide [1]. Adenocarcinoma represents 20–25% of cervical cancers with increasing incidence in recent years. Approximately 80–90% of cervical adenocarcinomas (ECA) are HPV-associated, and approximately 10–20% are unrelated to HPV infection (Fig. 1) [2–4]. Gastric-type endocervical adenocarcinoma (GAS) is the most common subtype of cervical non-HPV-associated carcinoma first described in 2007 by Japanese pathologists [5]. GAS is a very aggressive neoplasm with a five-year disease-specific survival rate of 30% compared with 77% in HPV- associated adenocarcinomas of the cervix [5]. These tumors often present at an advanced stage with a tendency for pelvic dissemination, specifically to the ovary, peritoneum, omentum, and distant metastases [6–9]. It displays a histomorphological spectrum from well- to poorly differentiated adenocarcinomas [10–12] and overlaps with HPV-associated (usual, mucinous, invasive stratified mucin producing carcinoma) adenocarcinoma of the cervix. Therefore, a precise diagnosis of this cervical adenocarcinoma variant is the key to adequate management. The objectives of this study were to determine the incidence of GAS in a single institution over the last 15 years and the clinical-pathological features of GAS compared to UEA with clinical outcomes.

## Materials and methods

This retrospective study was approved by the biomedical ethics review board of the University of Saskatchewan. Pathology reports with a diagnosis of adenocarcinoma of the cervix were obtained from the Saskatoon Health Region (SHR) pathology database from January 2000 through December 2015. We identified 159 cases with the search terminology “cervix adenocarcinoma” in the SHR database. Of these, 124 cases were adenocarcinoma in situ and 35 cases of invasive adenocarcinoma of the cervix. Slides and reports were available on all of these cases.

The slides were reviewed by two pathologists by simple and reproducible criteria suggested by International Endocervical Adenocarcinoma Criteria and Classification (IECC) into two groups, HPV- associated (HPVA) and non HPV associated (NHPVA) by morphologic criteria: easily identifiable apical mitotic figures and apoptotic bodies at scanning magnification as HPV-associated [4, 13]. The HPV associated lesions were further subclassified based on cytoplasmic features into HPVA-UEA -Mucinous adenocarcinoma, intestinal type, and signet-ring cell type. Criteria for Gastric-type adenocarcinoma included: tumor cells with abundant clear, foamy, or pale eosinophilic cytoplasm and distinct cytoplasmic borders. Minimal deviation adenocarcinoma was included as part of a spectrum of GAS. The histopathologic assessment included tumor size, grade, depth of invasion, lymph-vascular space invasion (LVSI), and stage. Representative blocks were selected for immunohistochemical staining. Clinical data were obtained by retrospective review of medical records.

Tissue microarray (TMA) was constructed from paraffin blocks with 6 mm cores in duplicate from two areas of the representative tumor. Immunohistochemistry (IHC) was performed using antibodies for MUC-6, estrogen receptor (ER), progesterone receptor (PR), p16, p53, CK7, CK20, and CEA in dilutions as optimized and validated for routine clinical use. Table 1 shows the list of immunohistochemistry (IHC) assays, primary antibodies, and conditions used for testing. Following readout criteria were applied: i) if more than 25% tumor cells had strong cytoplasmic staining, the tumor was considered positive for MUC-6, ii) p16 was designated as positive when there was diffuse and block-like nuclear and cytoplasmic staining; iii) p53 had “mutation –type” expression if more than 80% of nuclei stained strongly in the tumor cells, and iv) ER and PR were considered as positive if more than 10% of tumor cells showed nuclear staining, and v) CK7, CK20, and CEA were designated as positive if they showed more than borderline cytoplasmic staining.

**Table 1:** Showing antibody clones, antibody dilutions and detection system

Primary Antibody	Clone (Source)	Dilution	Instrument	Antigen Retrieval	Detection System
Estrogen Receptor (ER)	EP1 (Dako/Agilent)	1/50	Autostainer Link 48	High pH	Envision Flex
Progesterone Receptor (PR)	16 (Leica/Novocastra)	1/200	Autostainer Link 48	High pH	Envision Flex
p53	D0–7 (Dako/Agilent)	RTU	Autostainer Link 48	High pH	Envision Flex
MUC-6	MRQ-20 (Sigma-Aldrich)	1/100	Autostainer Link 48	High pH	Envision Flex
p16	E6H4 (CINtec® Histology, Ventana-Roche)	RTU	BenchMark ULTRA	CC1	OptiView
CK7	SP52 (Ventana-Roche)	RTU	BenchMark ULTRA	CC1	OptiView
CK20	SP33 (Ventana-Roche)	RTU	BenchMark ULTRA	CC1	OptiView

After initial reassessment of 35 invasive adenocarcinomas, 11cases were reclassified and excluded from our study for the following reasons: endometrial endometrioid adenocarcinoma [5], serous endometrial carcinoma [5], poorly differentiated carcinoma [1]. Twenty-four cases were confirmed as invasive adenocarcinoma of the cervix and were reclassified as 19 UEA/HPVA (79.2%) and 5 GAS (20.8%).

Group characteristics were summarized using mean ± standard deviation and counts (percent). Groups were compared using the Mann-Whitney U test and Chi-Square test. When appropriate, the Fisher Exact method was used. Survival time was summarized in months using median and 95% confidence intervals. The Kaplan-Meier estimator and Log Rank test were used to compare survival time between groups. Patients were censored at the last known observation. The analyses were conducted using IBM SPSS Statistics v26.0 and GraphPad Prism v8.4.3.

## Results

The clinical and pathological features of GAS and UEA/HPVA cases are compared in Table 2. The average age at the diagnosis for GAS was 61.6 years (range 43 to 87 years). The presenting clinical symptoms included: watery or bloody vaginal discharge, pelvic pain, heavy post-coital bleeding, irregular vaginal bleeding, and postmenopausal bleeding. The average age at the time of diagnosis for UEA was 44.6 years (range 26 to 62 years). The clinical presentation in this group of patients was very similar to the GAS cases, including asymptomatic patients.

**Table 2:** Comparison and differences between GAS and UEA regarding stage, age, lymph-vascular permeation, lymph node status and local/distant metastases at the time of diagnosis. All data presented as count (percent), unless otherwise specified

	GAS ***N*** = 5	UEA ***N*** = 19	***P***-Value
**FIGO**			
I	2 (40)	11 (57.9)	0.31^a^
II	1 (20)	8 (42.1)	
III	2 (40)	–	
IV	–	–	
**Age (years), mean ± SD**	61.6 ± 16.5	44.3 ± 9.0	0.015^a^
**Tumor Size (cm), mean ± SD**	3.8 ± 0.8	1.9 ± 1.2	0.004^a^
**LVI**			
Present	3 (60)	3 (15.8)	0.079^b^
Not Present	2 (40)	12 (63.2)	
Unknown	–	4 (21.1)	
**Regional Lymph Node Mets**			
Present	1 (20)	2 (10.5)	0.52^b^
Not Present	4 (80)	14 (73.7)	
Unknown	–	3 (15.8)	
**Abdominal Spread**	3 (60)	–	0.0049
**Other Mets**	2 (40)	–	0.0362
**Recurrence**			
Yes	3 (60)	7 (36.8)	0.63^b^
No	2 (40)	10 (52.3)	
Unknown	–	2 (10.5)	
**P16**			
Positive	–	16 (84.2)	0.043^c^
Negative	5 (100)	0 (0)	
Unknown	–	3 (15.8)	
**MUC-6**			
Positive	5 (100)	–	< 0.0001^c^
Negative	–	17 (89.5)	
Unknown	–	2 (10.5)	
**P53**			
Positive	3 (60)	–	0.0049^c^
Negative	2 (40)	17 (89.4)	
Unknown	–	2 (11.6)	

a – Mann Whitney U test,

b – Fisher Exact comparing Present vs Not Present or Unknown,

c – Fisher Exact comparing Positive vs Negative or Unknown,

### Histology

All the cases of UEA/HPVA demonstrated apical mitotic figures and apoptotic bodies at scanning magnification. Seventeen showed decreased cytoplasmic mucin (UEA), one with intracytoplasmic mucin (mucinous), and one with signet ring cell type cells.

**Fig. 2:**
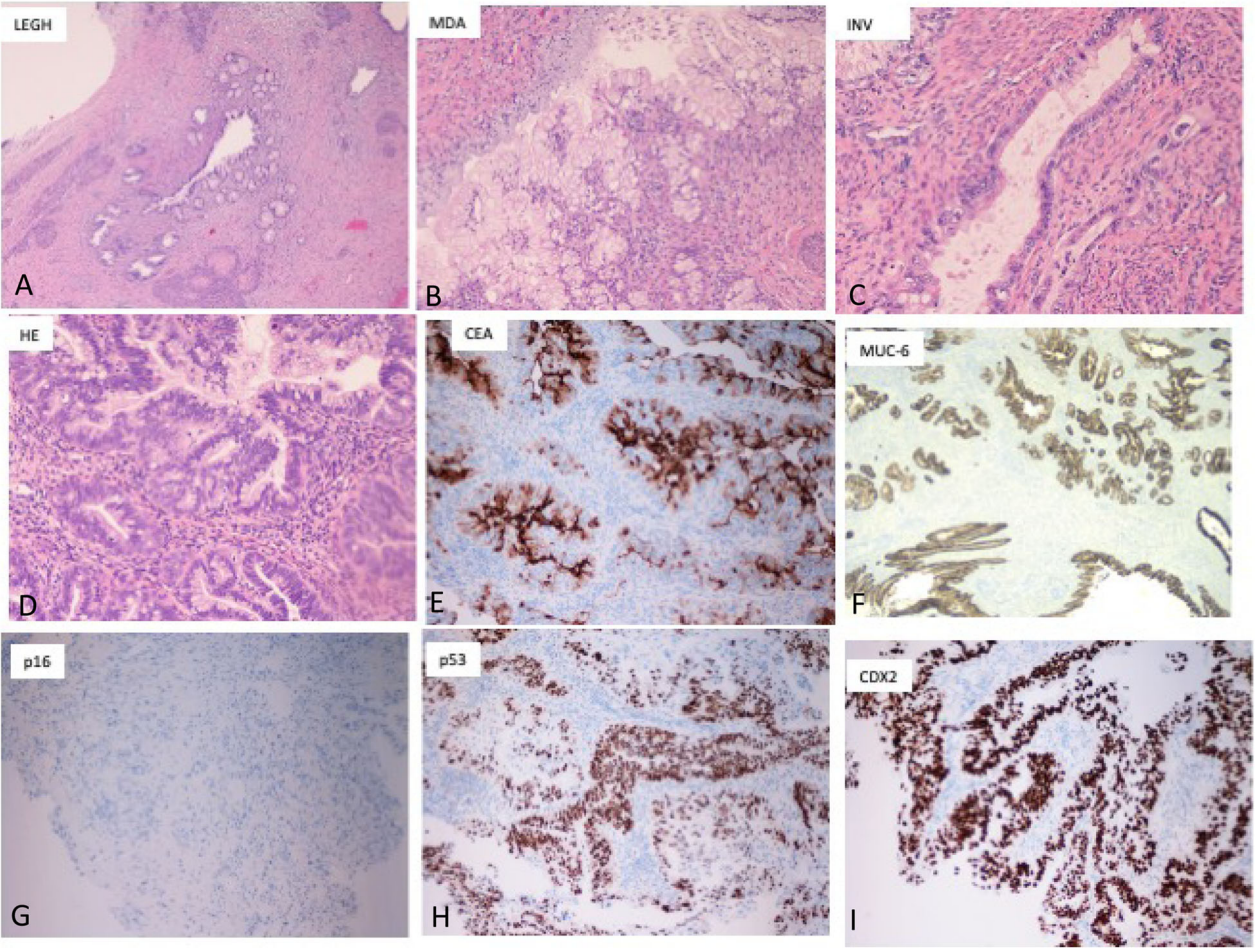
Images from well-differentiated MDA (B), arising on the base of LEGH (**A**) with centrally dilated duct surrounded by small proliferating glands. **B**. MDA with intraluminal papillary infoldings lined by columnar pale cells with abundant mucin, distinct cell borders and very mild nuclear enlargement. **C**. Focus on stromal invasion by single and small clusters of neoplastic cells. **D**. HE of moderately-differentiated GAS with columnar pale to eosinophilic cells with nuclear enlargement, stratification and hyperchromasia. Dispersed goblet cells are present Single images of IHC with different antibodies **E**. CEA, **F**. MUC-6 **G**.p16 **H**.p53 and **I**.CDX2

Five cases did not demonstrate the above features. Two of these cases were diagnosed as MDA on initial cervical biopsy and resection specimen. Both of these cases showed classic morphologic features of MDA as extremely well-differentiated, deeply infiltrating, deceptively bland endocervical glands, some with complex outlines. There were also foci of associated lobular endocervical glandular hyperplasia (LEGH). On the other hand, all three cervical biopsies of GAS were diagnosed as UEA [2] and one as serous carcinoma suggesting difficulties and lack of awareness of GAS diagnostic criteria. However, using IECC criteria, these cases were correctly diagnosed as GAS on review for this study. All these three cases of GAS demonstrated well-differentiated (MDA-like) to moderately differentiated areas with variable-sized, deeply infiltrating, and focally crowded neoplastic glands. The columnar epithelium showed voluminous pale eosinophilic cytoplasm, distinct cell borders with moderately enlarged slightly hyperchromatic round nuclei with irregular membranes. Rare mitotic figures and basal apoptotic bodies were noted on high magnification (Fig. 2).

### Immunohistochemistry

All the five cases of GAS were diffusely positive for CK7, MUC-6 (> 70% of tumor cells), three cases were strongly and diffusely positive for p53 (mutant type), and two showed a wild-type expression pattern for p53(normal pattern). Four of the cases stained negative for ER, PR and one showed focal, weak ER/PR positivity. Three cases were positive for CEA, and one case was positive for CDX2, supporting intestinal differentiation. Sixteen (out of 19) cases of UEA (84.2%) demonstrated diffuse, block-like nuclear and cytoplasmic positivity for p16 and seventeen cases were negative for CDX-2, MUC-6, and CK20. p53 demonstrated wild-type staining in 15 UEA cases. Three cases were focally positive for ER and one for PR (Table 2).

### Treatment and follow up

**Fig. 3:**
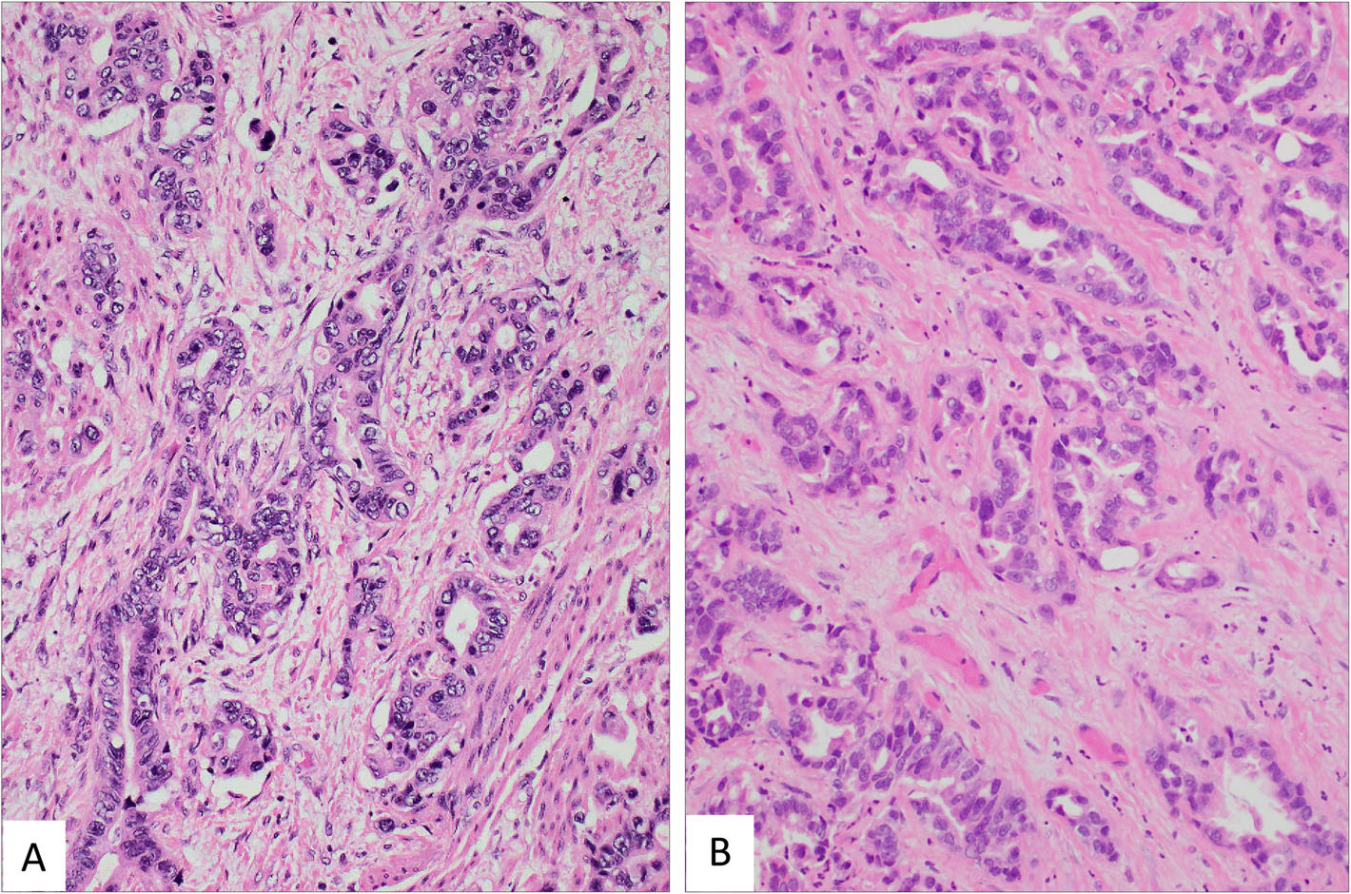
Primary tumor was composed mostly of irregular glands, nests of cells, and occasional invasive single cells. The nuclei varied in size and shape and there was prominent variation from vesicular to highly hyperchromatic nuclei. Nucleoli were present in many cells, but not in all. Occasional goblet cells were also present. Mitotic figures and apoptotic bodies were frequent. Hypocellular and edematous stroma was dominant in many areas of the tumor. The morphology is nearly identical in the primary tumor (**A**) and metastasis in skin (**B**) a year after the diagnosis

Local recurrence/metastasis occurred in 10 (3/5 GAS, 7/19 UEA) of the 24 patients (Table 3). Recurrent or metastatic GAS cases demonstrated similar histomorphologic features as primary tumor (Fig. 3). Five of nineteen UEA patients with recurrence were stage IIB, one stage IIA and one Stage IB, with two found to have a distant recurrence, two both local and distant recurrences, and three local recurrences with pelvic lymph node involvement died of renal complications. Two of the recurrent cases had UEA/HPVA -mucinous, NOS, and signet ring cell type adenocarcinoma, respectively. All the patients with local and distant recurrences received palliative treatment with either one or more lines of systemic chemotherapy or local palliative radiation and or both in accordance with national guidelines after being discussed in Multidisciplinary Gynecologic Oncology tumor rounds.

**Table 3:** Characteristics of treatment and follow up. All data presented as count (percent), unless otherwise specified

	GAS ***N*** = 5	UEA ***N*** = 19	***P***-Value
**Treatment**			
Radical Hysterectomy	4 (80.0)	12 (63.2)	0.63
Chemotherapy	2 (40.0)	3 (15.8)	0.27
Radiation Therapy	3 (60.0)	5 (26.3)	0.29
Pelvic Exenteration	1 (20.0)	0 (0.0)	0.21
LEEP	0 (0.0)	9 (47.4)	0.19
Cervical Biopsy	0 (0.0)	2 (10.5)	0.99
**Recurrence**			
Yes	3 (60.0)	7 (36.8)	0.61
Not Observed	2 (40.0)	12 (63.2)	
**Death**			
Yes	3 (60.0)	6 (31.6)	0.33
Not Observed	2 (40.0)	13 (68.4)	
**Time to Death (months)**			
Median (95% CI)	22.0 (15.6, 28.4)	118.0 (59.4, 176.6)	0.043^a^

a – Test of equality of survival distributions using the Kaplan-Meier Estimator and Log Rank test

**Fig. 4:**
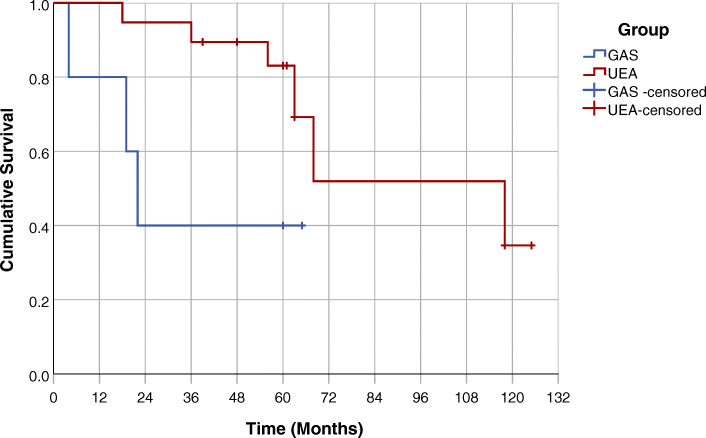
Kaplan-Meier Curve of the time to death in months among the GAS and UEA patients. Test of equality of the survival distributions was assessed using the Log Rank test (*p* = 0.043)

Death was observed in three GAS patients and six UEA patients. Time to death varied from 18 to 108 months, respectively. As shown in Fig. 4, the GAS group had a significantly shorter median survival time than the UEA group (GAS = 22.0 months [95% CI 15.6, 28.4] vs. UEA = 118.0 months [95% CI 59.4, 176.6], *p* = 0.043).

## Discussion

Adenocarcinomas of the cervix (ECA) account for approximately 20–25% of cervical carcinomas, of which 80–90% are related to HPV infection, and the remaining are NHPVA. HPVA/UEA patients are younger, usually present at an early stage, and have a better prognosis compared to NHPVA. Non-HPV-associated GAS patients are usually older, present at higher stage, have poor outcomes, and should be correctly recognized in cervical biopsy specimens for better patient management. ECAs are classified according to the WHO system predominantly based on cytomorphological features into more than ten different types and are confusing for both pathologists and clinicians [4]. Recently, IECC has validated the ECA classification system based on simple morphologic criteria into two etiologic groups: HPVA and NHPVA, using easily identifiable apical mitotic figures and apoptotic bodies at scanning magnification [13–16]. We found these criteria to be simple and reproducible. In this study, GAS was also the most common subtype of NHPVA, accounting for 20.8% of all ECA. The exact incidence of GAS in North America is not yet known, but larger multi-institutional international studies reported it as 10% and up to 20–25% in the Japanese population [5, 13, 17]. In this study, four GAS patient were Caucasian, and one was Aboriginal.

Malignant gastric-type cervical lesions comprise MDA and GAS. The patients usually present with watery discharge or enlarged cervix (barrel cervix -due to axially and radially infiltrating neoplastic glands). These lesions usually arise in the upper endocervix in contrast to UEA/HPVA, which arise in the transformation zone and present as a mass lesion. MDA is a well-differentiated morphologic spectrum of GAS. MDA usually shows very deceptively bland, deeply infiltrating glands in the wall of the cervix. Therefore, it is very challenging to establish a malignant diagnosis of MDA in superficial biopsies; thus, a high index of suspicion and radiological correlation is required. In our two MDA cases, the clinical history, enlarged cervix, and corresponding radiological findings, along with deep cervical biopsies, aided in guiding the histologic diagnosis. The histologic features/grading in MDA is important in reaching a correct diagnosis but has no impact on clinical outcomes. One of our cases presented at an early stage, while the second case had locally advanced disease at the time of diagnosis. Therefore the umbrella term GAS is recommended for these lesions [4–13].

The histologic features of GAS are still under-recognized. Three of our five cases were diagnosed as UEA or serous carcinoma on biopsy specimen due to under-recognition of histologic features of GAS and/or lack of routine use of p16 or misinterpretation of p53 staining for serous carcinoma. The key cytoplasmic features of GAS are similar to MDA [4, 5, 13]. However, nuclei may vary from uniform, round bland, basally located as noted in MDA to moderate, marked atypia with enlargement, and irregular cell membranes in moderately to poorly differentiated GAS [10, 12].

These cytological features of GAS overlap with HPVA-UEA -mucinous adenocarcinoma, endometrioid, endometrial and serous endometrial carcinoma [13–16]. IECC criteria are simple and reproducible to differentiate between HPVA and NHPVA-ECA. The diagnosis should be confirmed by immunohistochemistry for p16/HPV, MUC-6, and p53. P16 is a surrogate marker for HPV-related malignant lesions and is a useful adjunct in confirmation of UEA on cervical biopsy. If it is negative or shows a mosaic pattern, MUC-6 and p53 IHC may aid in the diagnosis of GAS. The immunohistochemical profile of GAS is similar to normal gastric mucinous cells with expression of MUC-6, HIK 1083. GAS cells produce neutral cytoplasmic mucin that stains red with combined Alcian blue/ periodic-acid-Schiff (PAS) stain, while normal endocervical glands cells/UEA contain an admixture of acid and neutral mucin that stains a purple-violet color with Alcian blue/PAS. Immunohistochemical markers, MUC-6 and HIK-1083, both detect pyloric gland mucin, and support gastric-type differentiation. In this study, all the GAS cases were MUC-6 positive and p16 negative. In the literature, there is a wide variability in MUC − 6 expression and varies from 40 to 81 to upto 100% [5, 14, 17–21]. Some of this variability may be due to methodology and or use of tissue microarray. In addition, MUC-6 is positive in approximately 8% of benign epithelial cells, normal endometrial glands, and significant number of endometrial endometrioid carcinomas [22]. In contrast, HIK-1083 stains only 2% of benign epithelial cells and negative in all types of endometrial carcinoma. These findings suggest a cautious interpretation of MUC 6 IHC to differentiate between benign and malignant mimics of GAS. HIK1083 has been reported to be more specific but less sensitive marker of pyloric gland mucin. However, HIK 1083 antibody has limited availability [13]. A significant number of GAS cases also show mutant type expression pattern with p53 immunostain (40–52% in larger studies) and 60% in this study implicating a role for p53 in pathogenesis of GAS [5, 14, 18–21].

Diagnosis of GAS is challenging, mainly in small cervical biopsies. Benign mimics of GAS, especially of MDA, includes Lobular endocervical glandular hyperplasia (LEGH), Deep nabothian cysts, diffuse laminar endocervical glandular hyperplasia and mesonephric hyperplasia. Lobular endocervical glandular hyperplasia (LEGH) is characterized by well demarcated proliferation of small to medium sized glands around a dilated central gland located in inner half of cervical wall. These glands are lined by tall mucinous epithelium with gastric type differentiation. LEGH is a part of spectrum of pyloric gland metaplasia and is considered to be non-obligatory precursor lesion of GAS. Approximately 75% of these lesions are positive for HIK-1083 and MUC-6. These lesion can be differentiated from GAS by superficial location, lobular arrangement, lack of cytologic atypia and focal desmoplasia as highlighted by smooth muscle actin (SMA). p53 immunostain may support a diagnosis of GAS if mutant type staining pattern is present [17, 23–25]. Deep nebothian cysts are usually present superficially but rarely may extend upto serosa and raise a diagnostic consideration of GAS. Nebothian cysts are lined by columnar to flattened endocervical type epithelium, filled with mucin and are devoid of atypical features, mitotic acvity and lack desmoplastic stroma. Benign endocervical epithelium is positive for ER and PR and this immunostain, along with SMA may help to differentiate deep nabothian cysts from well differentiated GAS [26]. Another, though rare, benign mimic of GAS is diffuse laminar endocervical glandular hyperplasia characterized by a laminar proliferation of closely packed endocervical glands, confined to inner third of cervical wall and sharply demarcated from adjacent stroma. These glands are lined by tall mucinous epithelium with round, bland basally located nuclei. These glands are round and regular but irregular glands may be observed. These glands are usuallu associated with acute and chronic inflammatory infiltrate. These are rare lesions and usually are incidental findings with few case series and case reports. These lesions can be differentiated from GAS based on the lack of symptoms, superficial location, sharply demarcated border and lack of cytologic atypia and desmoplasia [27, 28]. Immunostaining pattern of these benign lesions by MUC 6 and HIK 1083 remains unknown. Another challenging deep seated lesion that can be confused with GAS in a biopsy specimen is mesonephric remnant, or mesonephric hyperplasia. Mesonephric remnants are usually located in the lateral wall of cervix deep to normal endocervical glands. Mesonephric hyperplasia demonstrates lobular arrangement of small to medium sized, uniformly spaced tubules. Tubules are lined by cuboidal cells with scant cytoplasm and round to oval bland nuclei. Tubules typically contain dense, PAS-positive eosinophilic secretions. These lesions are usually positive for PAX2, BCL2, androgen receptor, GATA-3 and luminal pattern expression of CD10. MDA can be differentiated on morphologic basis as mesonephric hyperplasia that lacks mucinous epithelium with voluminous cytoplasm and desmoplastic stroma [29]. Malignant mimics of GAS include UEA/HPVA, endometrial endometrioid carcinoma, specifically mucinous type, and serous carcinoma. UEA/HPVA endocervical carcinomas including intestinal and signet ring type mucinous carcinomas can be differentiated from GAS based on IECC criteria and p16 immunostain usually shows diffuse block-like nuclear and cytoplasmic staining. GAS usually arises in the upper endocervix and may extend to the lower uterine segment. On cervical biopsy specimens, differential diagnosis of GAS also includes mucinous endometrial endometrioid adenocarcinoma. These are FIGO grade I adenocarcinomas, ER, PR positive with an excellent prognosis. ER, PR immunohistochemistry can differentiate between these two entities on the biopsy specimen. In one study, positive staining with MUC 6 was noted in significant number of endometrial endometrioid carcinoma and should be interpreted cautiously. HIK 1083 was negative in all the endometrial endometrioid carcinoma and future availability of HIK 1083 antibody will be very valuable to differentiate GAS from malignant mimics [22]. Approximately 50% of GAS cases are positive for p53 and must be differentiated from endometrial serous carcinoma, which is positive for p16, ER, and PR.

Our study showed a higher rate of recurrence and metastasis in GAS compared to other studies that reported recurrence in 31 to 45% of cases. This may be due to the small sample size of our study. The median survival among those with GAS was 22 months compared to 118 months among those with UEA. This is consistent with larger studies [6, 7, 16, 21]..The poorer outcome may be related to GAS being more resistant to both radiotherapy and chemotherapy. Our results also demonstrate that recurrent UEA/HPVA cases had a better response to adjuvant chemotherapy and radiotherapy compared to patients with GAS.

In conclusion, this study highlights the importance of awareness of GAS, its diagnostic dilemmas and the need to recognize clinical, morphological features of GAS, the significance of IECC criteria to differentiate between HPVA and non-HPV associated ECA, and the role of p16, MUC-6, ER, PR and p53 immunohistochemistry in differential histopathological diagnosis. GAS usually presents at an advanced stage and therefore, ovarian conservation may not be recommended even in younger patients, and omentectomy could be considered as part of surgical management. The cause of aggressive natural history remains unknown, maybe due to lack of early detection on PAP smears, diagnostic challenges, and resistance to therapy. ECA are heterogeneous tumors with different etiologies and driver mutations but are being treated with a universal approach. There is a need for further studies to characterize novel systemic agents in addition to traditional chemotherapy drugs or optimal radiotherapy than similar protocols used for recurrent UEA.

## Data Availability

All data generated or analyzed during this study are included in this manuscript.

## Abbreviations

ECA: Cervical adenocarcinoma
HPVA: HPV-associated
NHPVA: Non-HPV-associated
HPVA- UEA or UEA: Human papillomavirus-associated usual type endocervical adenocarcinoma
GAS: Gastric-type endocervical adenocarcinoma
IECC: International endocervical adenocrcinoma criteria and classification
LVSI: Lymph-vascular space invasion
IHC: Immunohistochemistry
TMA: Tissue microarray
MDA: Minimal deviation adenocarcinoma
LEGH: Lobular endocervical glandular hyperplasia
ER: Estrogen receptor
PR: Progesterone receptor
MUC 6: Gastric mucin 6
P16: p16^INK4a^, cyclin-dependent kinase inhibitor 2A
P53: Protein p53
